# MST4 negatively regulates type I interferons production via targeting MAVS-mediated pathway

**DOI:** 10.1186/s12964-022-00922-3

**Published:** 2022-07-12

**Authors:** Wei Liu, Zhenling Ma, Yaru Wu, Cui Yuan, Yanyan Zhang, Zeyang Liang, Yu Yang, Wenwen Zhang, Pengtao Jiao

**Affiliations:** 1 College of Life Sciences, Henan Agricultural University, Zhengzhou, 450002 China; 2 CAS Key Laboratory of Pathogenic Microbiology and Immunology, Institute of Microbiology, Chinese Academy of Sciences, Beijing, 100101 China

**Keywords:** MST4, Innate immunity, RIG-I, Type I interferons, MAVS

## Abstract

**Background:**

Cytosolic RNA sensing can elicit immune responses against viral pathogens. However, antiviral responses must be tightly regulated to avoid the uncontrolled production of type I interferons (IFN) that might have deleterious effects on the host. Upon bacterial infection, the germinal center kinase MST4 can directly phosphorylate the adaptor TRAF6 to limit the inflammatory responses, thereby avoiding the damage caused by excessive immune activation. However, the molecular mechanism of how MST4 regulates virus-mediated type I IFN production remains unknown.

**Methods:**

The expression levels of IFN-β, IFIT1, and IFIT2 mRNA were determined by RT-PCR. The expression levels of p-IRF3, IRF3, RIG-I, MAVS, and MST4 proteins were determined by Western blot. The effect of secreted level of IFN-β was measured by ELISA. The relationship between MST4 and MAVS was investigated by immunofluorescence staining and coimmunoprecipitation.

**Results:**

In this study, we reported that MST4 can act as a negative regulator of type I IFN production. Ectopic expression of MST4 suppressed the Poly (I:C) (polyino-sinic-polycytidylic acid)- and Sendai virus (SeV)-triggered production of type I IFN, while the knockdown of MST4 enhanced the production of type I IFN. Mechanistically, upon SeV infection, the MST4 competed with TRAF3 to bind to the 360–540 domain of MAVS, thereby inhibiting the TRAF3/MAVS association. Additionally, MST4 facilitated the interaction between the E3 ubiquitin ligase Smurf1 and MAVS. This promoted the K48-linked ubiquitination of MAVS, thereby accelerating the ubiquitin-mediated proteasome degradation of MAVS.

**Conclusions:**

Our findings showed that MST4 acted as a crucial negative regulator of RLR-mediated type I IFN production.

**Video Abstract**

**Supplementary Information:**

The online version contains supplementary material available at 10.1186/s12964-022-00922-3.

## Background

The innate immune reactions against pathogen invasion are mediated by the host pattern-recognition receptors (PRR), including RIG-I-like receptors (RLRs), toll-like receptors (TLRs), nucleotide-binding oligomerization domain-like receptors (NLRs), C-type lectin receptors, and PYRIN family members [1, 2]. RIG-I-like receptors (RLR) belong to the family of cytoplasmic RNA helicases such as retinoic acid-inducible gene I (RIG-I), melanoma differentiation-associated gene 5 (MDA5), and laboratory of genetics and physiology gene 2 (LGP2). RLR functions as the cytosolic viral RNA sensor and is involved in restricting viral replication and dissemination [3]. RIG-I senses viral RNA in the cytoplasm and triggers immune signaling through downstream mitochondrial antiviral signaling protein (MAVS) [4]. The complex formation of RIG-I-MAVS transduces the antiviral signal to downstream kinases, including TBK1/IKKε and IKKα/β, which further activates the transcriptional factors IRF3 and NF-κB, respectively, thus, stimulating the expression of type I interferon (IFN) and proinflammatory cytokines [5]. Type I IFN leads to the expression of hundreds of interferon-stimulated genes (ISGs), producing an antiviral state that invokes innate and adaptive immune cells to eliminate infection [6].

The production of type I interferon and proinflammatory cytokines must be tightly regulated since aberrant production of type I interferon can be harmful or even fatal to the host [7]. Thus, it is extremely crucial to prevent the deleterious overproduction of type I IFNs for maintaining homeostasis and to impede the progression of diseases, such as the outcomes of COVID-19 [8, 9]. The MAVS and TRAF3 are the key components involved in type I IFN production, and their activities are tightly regulated to maintain the balance of immune homeostasis. Multiple molecules act as negative regulators of type I IFN production by regulating the stability of MAVS along with the formation of the MAVS/TRAF3 complex in a variety of manners. For instance, the E3 ubiquitin ligase RNF114 inhibits the innate immune response against the red-spotted grouper nervous necrosis viral infection by targeting the interaction between TRAF3 and MAVS in sea perch [10]. The hereditary hemochromatosis protein HFE inhibits the production of type I IFN signaling by targeting the SQSTM1-mediated autophagic degradation of MAVS [11]. Moreover, the SARS-CoV-2 ORF10 suppresses the antiviral innate immune response by degrading MAVS through mitophagy [12].

Mammalian sterile 20-like kinase 4 (MST4; also known as STK26) is a ubiquitously expressed and highly conserved serine/threonine kinase in the MST family [13]. MST4 is a critical regulator of various cellular processes, including signal transduction, cell proliferation, apoptosis, cell-cycle control, cancer metastasis, and cell migration [14–16]. The MST4 is known to mediate cell growth and transformation via modulating the ERK signaling pathway, which is responsible for promoting the progression of prostate cancer and cancer metastasis [17]. Besides its expression dynamically responding to bacterial infection, MST4 can also directly phosphorylate the adaptor TRAF6 to limit the inflammatory responses, thereby avoiding the damage caused by excessive immune activation [18]. Also, MST4 stimulates autophagy via the phosphorylation-dependent activation of ATG4B in glioblastoma [19]. These findings indicate a potential negative function of MST4 in autoimmunity and host defense.

In the present study, we sought to characterize the function of MST4 in the innate immune response and discovered that the potent negative regulation of type I IFN signaling was done by MST4. MST4 strongly hindered the type I IFN production by impairing the TRAF3/MAVS association and facilitating the Smurf1-mediated K48-linked ubiquitination of MAVS, thereby resulting in its degradation and subsequent inactivation of downstream signaling pathways. Therefore, our study provides molecular insights for MST4-driven regulation of cytosolic RNA sensing, revealing a new physiological function of MST4 in innate antiviral immunity.

## Materials and methods

### Reagents, antibodies, and cells

TRIzol (Cat#15596-026; Invitrogen, Carlsbad, CA, USA), and TB Green (Cat#R820A; Takara, Tokyo, Japan) were purchased from the indicated manufacturers; M-MLV (Cat#M1701) and luciferase assay kit (Cat#E1500) were purchased from Promega (Madison, WI, USA); cycloheximide (CHX, Cat#66819), Poly (I:C) (Cat#P1530), and DAPI (Cat#D9542) were from Sigma (St. Louis, MO, USA).

For immunoblot analysis, the following antibodies were used: anti-MST4 (1:1000, Cat#, ab52491) was obtained from Abcam (Cambridge, MA, USA); anti-c-Myc (1:2000, Cat#C3956), anti-MAVS (1:1000, Cat#SAB1400655), and anti-Flag M2 (1:2000, Cat#F3165) were obtained from Sigma (St. Louis, MO, USA); anti-HA (1:2000, Cat#2999), anti-IRF3 (1:1000, Cat#4302), anti-TRAF3 (1:1000, Cat# 61095), and anti-K48-linkage specific polyubiquitin (1:1000, Cat#4289) were obtained from Cell Signaling Technology (Danvers, MA, USA); anti-GAPDH (1:2000, Cat#sc-25778), anti-mouse IgG (1:5000, Cat#sc-137075), and anti-rabbit IgG (1:5000, Cat#sc-2357) were obtained from Santa Cruz Biotechnology (Santa Cruz, CA, USA).

### Cell culture, Poly (I:C) transfection and virus infection

HEK293T cells were cultured in Dulbecco’s Modified Eagle’s Medium (GIBCO, Grand Island, NY, USA) supplemented with 10% fetal bovine serum (FBS, GIBCO), 100 U/mL penicillin, and 100 mg/mL streptomycin at 37 °C with 5% CO_2_. For Poly (I:C) transfection, 293 T cells cultured in 6-well plates were transiently transfected with 10 μg/mL Poly (I:C) using Lipofectamine 2000 (Cat#11668019; Invitrogen, Carlsbad, CA) according to the manufacturer’s instructions. Sendai virus (SeV) was provided by professor Wenjun Liu (Institute of Microbiology, Chinese Academy of Sciences, China). HEK293T cells were infected with SeV at a multiplicity of infection (MOI) of 1 for 8 h and collected for further analysis.

### shRNA and siRNA

To generate MST4 knockdown cell lines, we infected HEK293T cells with a recombinant lentivirus carrying GFP and sh-MST4. The procedures of lentivirus transfection were performed as previously described [20]. The small hairpin RNA (shRNA) (Cat#30323; Addgene, Cambridge, MA, USA) target sequences used were as follows: sh-MST4 1# (5′-GCTGCCAATGTCTTGCTCTCA-3′), sh-MST4 2# (5′-GCTGGTCAGCTGACAGATACA-3′). HEK293T cells were transfected with lentiviral vectors encoding the shRNA while an empty vector was used as the negative control and was designated as lentiviral vector control. Supernatants containing lentivirus were harvested at 36–48 h after transfection and used to infect the target cells for 24 h. Stably transduced cells were selected in media containing 5 μg/mL puromycin (Cat#58582, Invivogen, San Diego, CA, USA). The knockdown efficiency was analyzed by immunoblot analysis.

### Plasmids

Sequences encoding human MST4 or IKKε were constructed into pcDNA3.1-Flag (Cat#V79520; Invitrogen, Carlsbad, CA, USA) or pCMV-Myc (Cat#631604; Clontech, Mountain View, CA, USA) vector with a Flag-tagged or Myc-tagged at the N terminus. MST4 KR, MST4 TE or Flag-tagged deletion constructs of MST4 was synthesized by GENEWIZ and then cloned into pCMV-Myc or Flag-tagged vector. All constructs were confirmed by DNA sequencing. IFN-β-Luc, NF-κB-Luc, ISRE-Luc, Flag-MAVS, Flag-TBK1, Flag-RIG-I, Flag-MDA5, Flag-RIG-I-N, Flag-IRF3, Flag-IRF3/5D, Flag-TRAF3, Flag-AIP4, Flag-Smurf1, HA-Ub, HA-K48-Ub, HA-K63-Ub, and HA-tagged deletion constructs of MAVS were previously described [21, 22].

### Transfection and reporter gene assay

Cells (1.5 × 10^5^) from different cell lines were transfected in a 24-well plate with IFN-β firefly luciferase and β-Gal plasmid together with other plasmids containing genes of interest using Lipofectamine 2000 (Cat# 11668019; Invitrogen, Carlsbad, CA, USA). Twenty-four hours later, cells were lysed in lysis buffer. After centrifugation at 12,000 g for 15 min at 4 °C, the supernatants were stored at − 80 °C. Luciferase assays were performed using a luciferase assay kit (Cat#E1500; Promega, Madison, WI, USA).

### Enzyme-linked immunosorbent assay

Cell culture supernatants were collected and assayed for cytokines. Cytokine production was measured by enzyme-linked immunosorbent assay of IFN-β according to the protocol of the manufacturer (Cat#DIFNB0; R&D Systems, Minneapolis, MN, USA).

### Subcellular fractionation

Cells (5 × 10^7^) infected with SeV or uninfected, were washed with PBS and lysed using a Nuclear and Cytoplasmic Protein Extraction Kit (Beyotime Biotechnology, Shanghai, China) according to the manufacturer’ s instructions.

### Indirect immunofluorescence

Cells were washed with PBS three times, fixed in 4% paraformaldehyde for 30 min at room temperature, permeabilized with 0.5% Triton X-100 in PBS (PBST) for 20 min, and stained with appropriate antibodies. Cell nuclei were stained with 5 μg/ml DAPI (Sigma, St. Louis, MO, USA). Following staining, cover slips were analyzed using a Leica SP8 confocal microscope.

### Quantitative PCR (qPCR) analysis

Cells were lysed, and total RNA was extracted using Trizol reagent. cDNA was made from total RNA using oligo (dT) primers with a a RevertAid First Strand cDNA Synthesis Kit (Cat# K1621; Fermentas, Waltham, MA, USA) according to the manufacturer’s instructions. Quantitative real-time PCR was performed using the TB Green and StepOnePlus PCR system (Applied Bio-systems). The following conditions were used for amplification of fragments: 95 °C for 30 s, followed by 40 cycles of 95 °C for 5 s, and 60 °C for 30 s. Relative quantification was expressed as 2^−Ct^, where –Ct is the difference between the main Ct value of triplicates of the sample and that of an endogenous GAPDH mRNA control. The Gene-specific primer sequences used are listed in the Supplemental Material (Additional file 1: Table S1).

### Coimmunoprecipitation and immunoblot analysis

Cells were lysed on ice for 30 min in lysis buffer containing 1% NP40, 150 mM NaCl, 10 mM Tris–HCl (pH 8.0), 10% glycerol, 1 mM EDTA, and protease inhibitor cocktail. After centrifugation, supernatants were incubated with anti-Flag sepharose affinity gel (Cat#A2220; Sigma, St. Louis, MO, USA) or with Protein A/G PLUS-Agarose beads (Cat#sc-2003; Santa Cruz, Santa Cruz, CA, USA) at 4 °C overnight. After five washes in wash buffer (1% NP40, 300 mM NaCl, 10 mM Tris–HCl (pH 8.0), 10% glycerol, and 1 mM EDTA), the immunoprecipitants were analyzed by immunoblotting. For western blot analysis, equal amounts of cell lysates and immunoprecipitants were resolved on a 10–12% sodium dodecyl sulfate polyacrylamide gel electrophoresis and then transferred to a PVDF membrane (Cat#ISEQ00010; Millipore, Billerica, MA, USA). After incubation with primary and secondary antibodies, the membranes were visualized by ECL chemiluminescence (Cat#32106; Thermo Fisher Scientific, Waltham, MA, USA).

### Data collection and statistical analyses

All experiments were repeated at least three independent batch and each batch was done at least three biological replicates, the averages and standard errors of all data shown were calculated from three biological replicates of one batch experiment. Data are expressed as mean ± SD. Statistical analyses were performed using Prism 5 software (GraphPad Prism, Version X; La Jolla, CA, USA). Statistics were calculated using Student’s t-test. *P* < 0.05 was considered statistically significant.

## Results

### MST4 negatively regulates type I IFN production

**Fig. 1:**
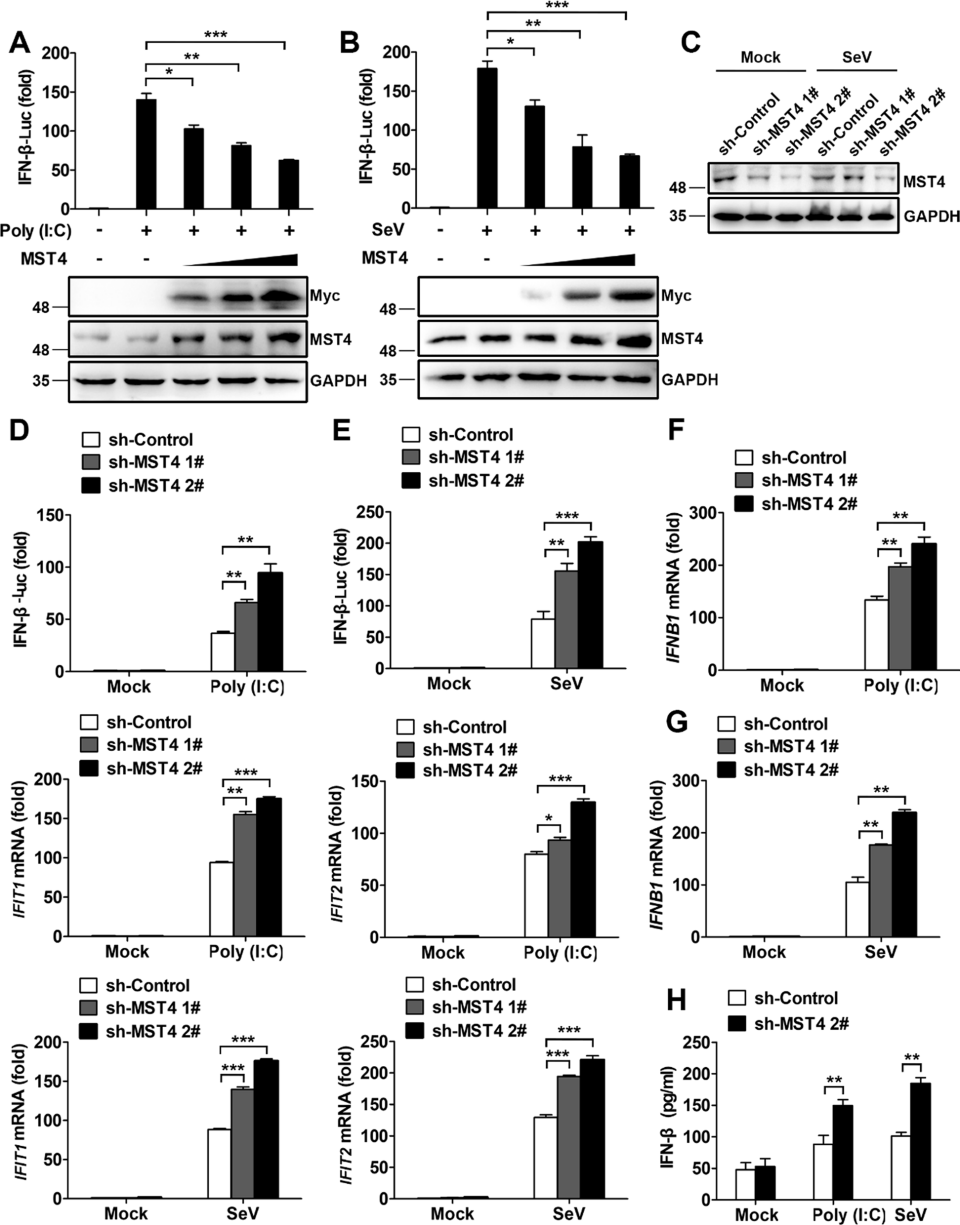
MST4 negatively regulates the production of RLR-mediated type I IFNs. **A**, **B** Luciferase activity in the lysates of 293 T cells transfected with IFN-β luciferase reporter (IFN-β-Luc), β-gal, and increasing concentrations of the Myc-MST4 expression plasmid (0, 50, 100, 200 ng) for 24 h and then treated or untreated with Poly (I:C) transfection (**A**) or SeV infection (**B**) for 8 h. Results are presented compared to the luciferase activity in the control cells treated with a luciferase reporter and empty vector. **C** Immunoblot analysis of MST4 in 293 T cells infected with lentiviruses carrying sh-MST4 1# and sh-MST4 2# (performed to generate stable cell lines), followed by infection with SeV for 8 h. **D**, **E** Luciferase activity in the lysates of 293 T-sh-Control and 293 T-sh-MST4 cells transfected with IFN-β-Luc, and transfected with Poly (I:C) (**D**) or infected with SeV (**E**) for 8 h. **F**, **G** Quantitative PCR analysis of the mRNA levels of *IFNB1*, *IFIT1*, and *IFIT2* in 293 T-sh-Control and 293 T-sh-MST4 cells transfected with Poly (I:C) (**F**) or infected with SeV (**G**) for 8 h. **H** ELISA results of IFN-β production in the supernatants of 293 T-sh-Control and 293 T-sh-MST4 cells transfected with Poly (I:C) or infected with SeV for 12 h. Data information: The data are represented as mean ± SD (**A**, **B**, **D**–**H**: n = 3). *P < 0.05, **P < 0.01, and ***P < 0.001 (unpaired two-tailed Student’s *t*-test). The data are representative of at least three independent experiments

To investigate the potential role of MST4 in the production of RLR-mediated type I interferons (IFN), the luciferase reporter assays were performed. The results showed that ectopically expressed MST4 strongly inhibited the reporter activation induced by Poly (I:C) (polyinosinic-poly-cytidylic acid) transfection and Sendai virus (SeV) infection in a dose-dependent manner (Fig. 1A, B). Consistently, we also found that MST4 hindered the IFN-β production at both protein and mRNA levels induced by Poly (I:C) transfection and SeV infection in 293 T cells (Additional file 1: Fig. S1A, B). IFNs stimulate the expression of interferon-stimulated genes (ISGs), which collectively inhibit viral replication. Therefore, we next examined the role of MST4 in regulating the expression of ISGs, such as *IFIT1* and *IFIT2* (encoding ISG56 and ISG54, respectively). As shown in Additional file 1: Fig. S1C, the ectopic expression of MST4 downregulated the mRNA levels of *IFIT1* and *IFIT2* induced by Poly (I:C) transfection and SeV infection, which suggested that MST4 inhibited IFN production induced by Poly (I:C) and SeV in 293 T cells.

To evaluate whether the knockdown of MST4 favored the activation of IFN-β, we generated MST4 knockdown in 293 T cells using two short hairpin RNAs (shRNAs) targeting MST4 (Fig. 1C). Figure 1D–H demonstrate IFN-β promoter-driven luciferase assay, quantitative PCR assay, and ELISA assay results in 293 T cells in response to transfected Poly (I:C) or infection of SeV, respectively. The absence of MST4 remarkably increased the production of type I IFNs and ISGs. We also obtained similar results with other cell types, such as in THP-1 cells (Additional file 1: Fig. S1D–F) triggered by SeV. After the treatment with the shRNA, ‘rescue’ of the cells by transfection was performed to demonstrate that MST4 inhibited Poly (I:C) transfection and SeV-induced IFN-β-responsive luciferase activity (Additional file 1: Fig. S1G, H). Overall, our data indicated that MST4 negatively regulated type I IFN production. Since sh-MST4 1# and sh-MST4 2# had similar functions in RLR-mediated type I IFN production, we focused our study on sh-MST4 2#, which will hereafter be referred to as sh-MST4.

### MST4 suppresses the activation of IRF3 signaling pathways

**Fig. 2:**
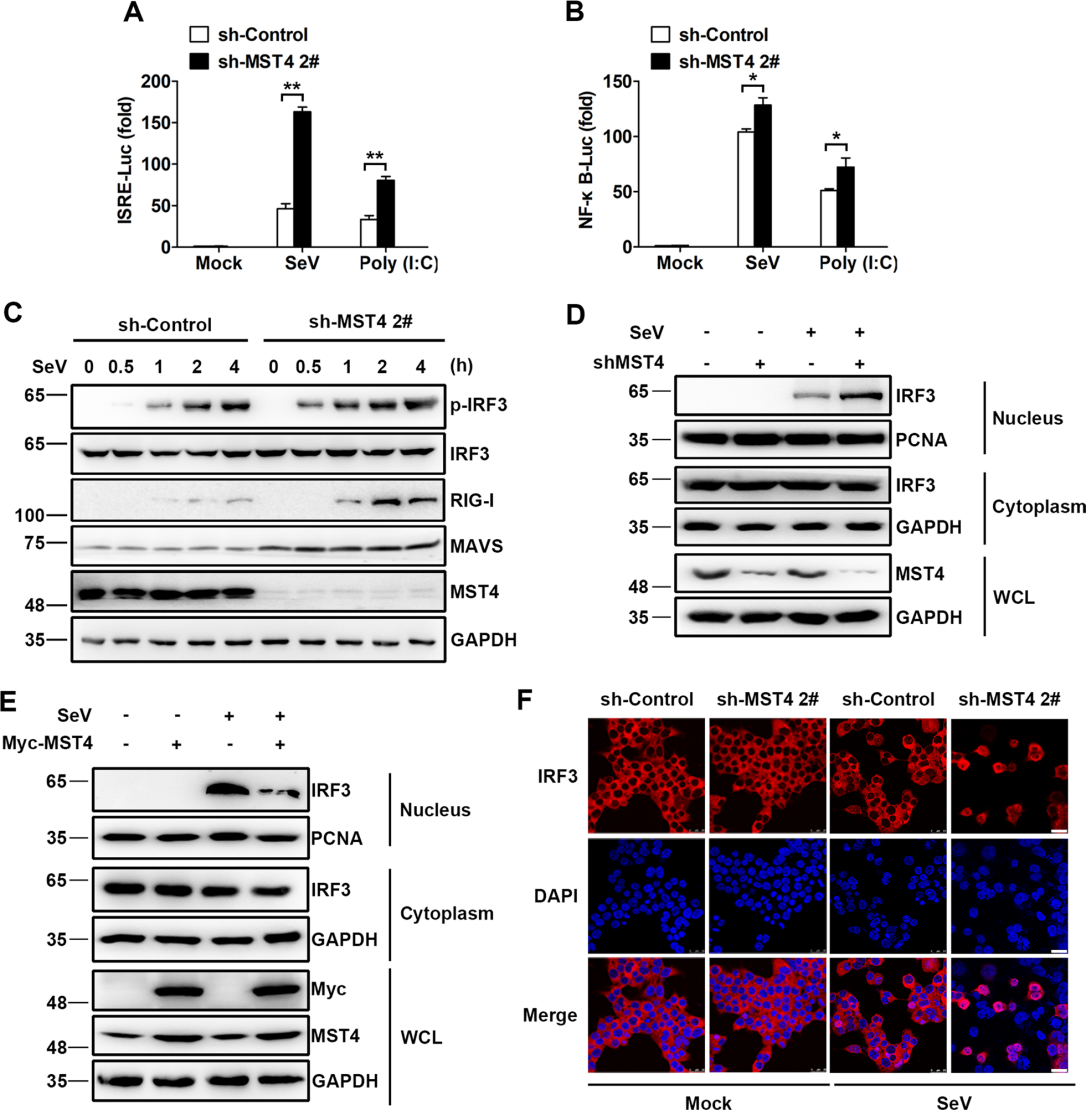
MST4 deficiency enhances the activation of IRF3. **A**, **B** Luciferase activity of lysates of 293 T-sh-Control and 293 T-sh-MST4 cells transfected with either ISRE-Luc (**A**) or NF-κB-Luc (**B**), plus β-gal for 24 h, followed by the infection with SeV or transfection with Poly (I:C) for 8 h. **C** Immunoblot analysis of the indicated proteins in 293 T-sh-Control and 293 T-sh-MST4 cells infected with SeV at the indicated time points. **D** Immunoblot analysis of the indicated proteins in 293 T-sh-Control and 293 T-sh-MST4 cells infected with SeV for 8 h, followed by the nuclear-cytoplasm extraction. **E** Immunoblot analysis of the indicated proteins in 293 T cells transfected with Myc-MST4 or control vector treated with SeV for 8 h, followed by the nuclear-cytoplasm extraction. **F** Confocal microscopy of endogenous IRF3 in 293 T-sh-Control and 293 T-sh-MST4 cells infected with SeV for 8 h. Scale bars, 25 µm. Data information: The data are represented as mean ± SD (**A**, **B**: n = 3). **P < 0.01 (unpaired two-tailed Student’s *t*-test). The data are representative of at least three independent experiments

Expression of the IFN-β-mRNA requires the coordinated actions of two major transcription factors, IRF3 and NF-κB [23]. To investigate the effect of MST4 on Poly (I:C) and SeV-induced activation of IRF3 and NF-κB, we co-transfected the interferon-stimulated response element (ISRE), and NF-κB luciferase reporter constructs with sh-MST4 or control shRNA in 293 T cells. We found that ISRE- and NF-κB-responsive luciferase activity induced by SeV infection or Poly (I:C) transfection was dramatically higher in the absence of MST4 (Fig. 2A, B). Consistently, the overexpression of MST4 strongly impaired the SeV-induced activation of IFN-β, ISRE, and NF-κB promoters in a dose-dependent manner (Additional file 1: Fig. S2A–C).

IRF3 is a critical interferon regulatory factor, whose activation can drive the transcription of IFN-β. The activation hallmarks of IRF3 are phosphorylation and nuclear translocation [24]. In line with the luciferase activity, the phosphorylation of IRF3 was also found to be increased in the SeV-infected 293 T cells knocked down with MST4 compared to the control 293 T cells (Fig. 2C). After activation, phosphorylated IRF3 translocated to the nucleus from the cytoplasm and induced the expression of type I IFN genes. The effect of MST4 on the nuclear translocation of IRF3 was analyzed by Western blot and confocal microscopy. As shown in Fig. 2D, the nuclear translocation of IRF3 was increased in the absence of MST4 after SeV infection, while the level of nuclear-translocated IRF3 was significantly reduced following the expression of MST4 (Fig. 2E). Consistently, confocal microscopy experiments indicated that the nuclear translocation of IRF3 was increased in the absence of MST4 after SeV infection (Fig. 2F).

### MST4 negatively regulates RLR-mediated type I IFN production

**Fig. 3:**
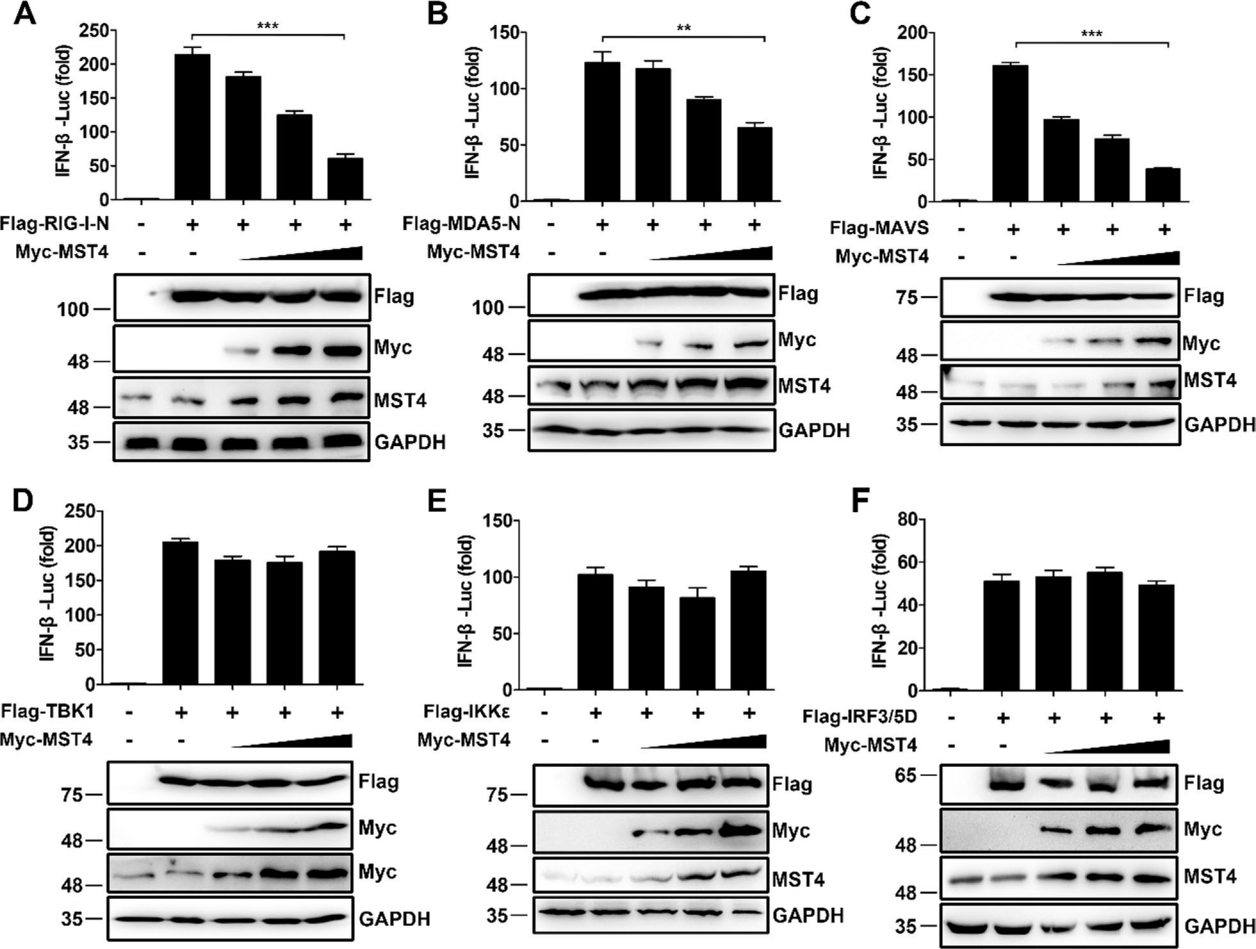
MST4 inhibits the production of type I IFN by targeting MAVS. **A**–**F** 293 T cells transfected with IFN-β-Luc and β-gal, along with the plasmid expressing Flag-tagged RIG-I-N (**A**), MDA5-N (**B**), MAVS (**C**), TBK1 (**D**), IKKε (**E**), or IRF3–5D (**F**), as well as increasing amounts of the plasmid encoding MST4. The expression levels of indicated proteins were analyzed by Western blot. Data information: The data are represented as mean ± SD (**A**–**F**: n = 3). **P < 0.01 and ***P < 0.001 (unpaired two-tailed Student’s *t*-test). The data are representative of at least three independent experiments

To determine the level at which MST4 exerted its inhibitory effect on RLR-mediated type I IFN production, we examined the effects of MST4 on the activation of IFN-β promoters mediated by various adaptors. The result showed that MST4 strongly inhibited the IFN-β reporter activation induced by RIG-I-N, MDA5-N, and MAVS in a dose-dependent manner (Fig. 3A–C) but not the activation induced by TBK1, IKKε, and IRF3/5D (a constitutively active form of IRF3; Fig. 3D–F). In agreement with these results, knockdown of MST4 showed enhanced activation of the ISRE and NF-κB induced by the overexpression of RIG-I-N, MDA5-N, and MAVS (Additional file 1: Fig. S3A, B). Moreover, similar results were observed during the expression of endogenous *IFNB1* mRNA induced by RIG-I-N, MDA5-N, and MAVS in the MST4 knockdown but not in the ones induced by TBK1 and IRF3/5D (Additional file 1: Fig. S3C). Overall, these results indicated that MST4 negatively regulates RLR-mediated type I IFN production.

### The interaction between MST4 and MAVS

**Fig. 4:**
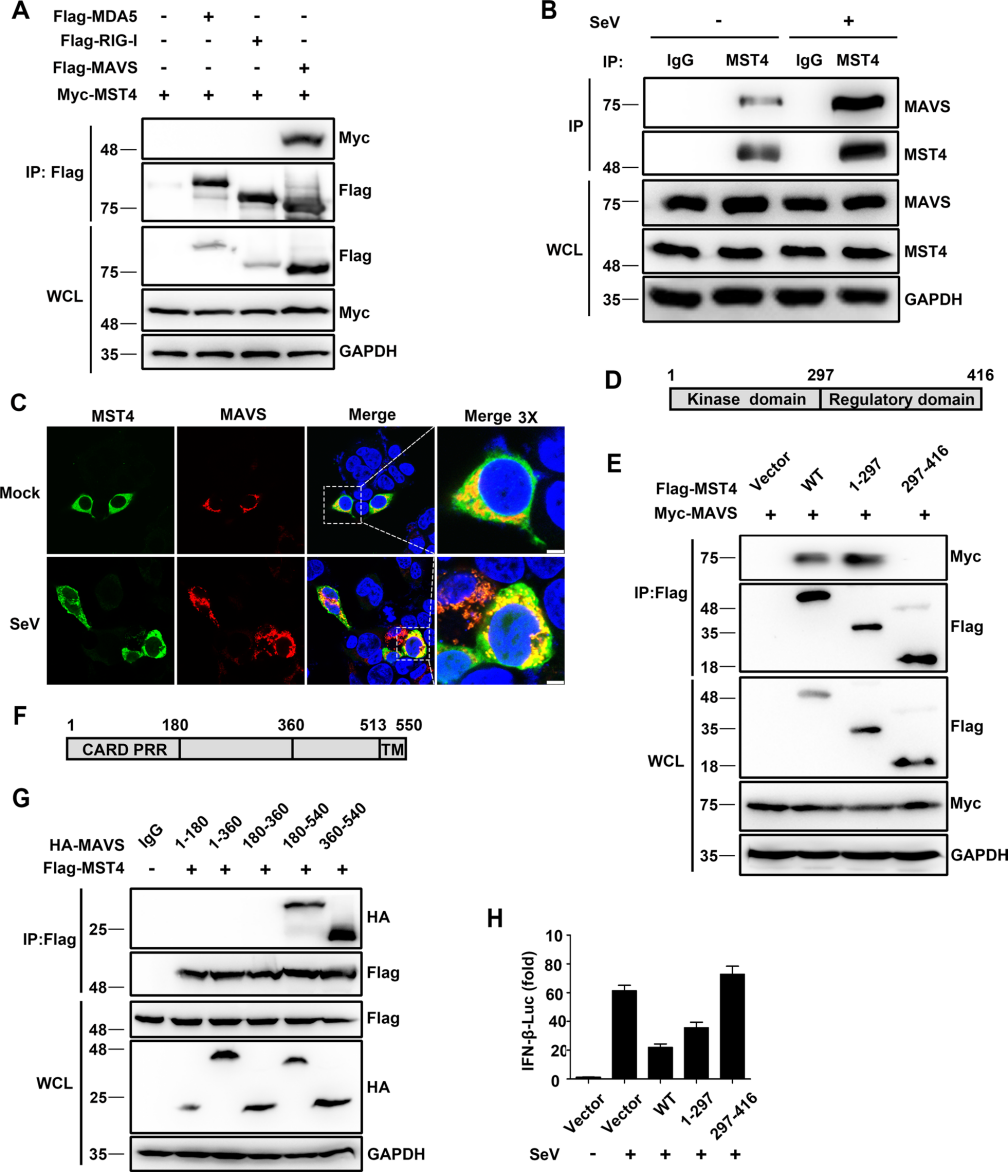
The interaction between MST4 and MAVS. **A** Immunoblot analysis of lysates of 293 T cells transfected with Flag-MDA5, Flag-RIG-I, or Flag-MAVS along with Myc-MST4 for 24 h, followed by immunoprecipitation with anti-Flag beads. **B** Immunoblot analysis of lysates of 293 T cells infected with SeV for 8 h, followed by immunoprecipitation with control rabbit-IgG or anti-MST4 antibodies. Lysates and immunoprecipitation extracts were probed with MST4 and MAVS antibodies. **C** Confocal microscopy of endogenous MST4 and MAVS in 293 T cells treated with SeV for 8 h. Scale bars, 10 µm. **D** Schematic representation of full-length MST4 and its truncated mutants. **E** Immunoblot analysis of lysates of 293 T cells transfected with Myc-MAVS and Flag-MST4 or its truncated mutants for 24 h, followed by immunoprecipitation with anti-Flag beads. **F** Schematic representation of full-length MAVS and its truncated mutants. **G** Immunoblot analysis of lysates of 293 T cells transfected with Flag-MST4 and HA-MAVS or its truncated mutants for 24 h, followed by immunoprecipitation with anti-Flag beads. **H** Luciferase activity in lysates of 293 T cells transfected with IFN-β-Luc and β-gal, along with Flag-MST4 or its truncated mutants, followed by the treatment with SeV for 8 h. Data information: The data are represented as mean ± SD (**H**: n = 3) and are representative of at least three independent experiments

To examine whether MST4 interacted with MDA5, RIG-I, or MAVS, the immunoprecipitation assays were performed in the 293 T cells, and Fig. 4A showed that MST4 could interact with the transfected MAVS. We further observed the endogenous interaction between MST4 and MAVS in the SeV-infected conditions (Fig. 4B). Confocal microscopy showed that MST4 was found in the cytosol, but MAVS was localized to mitochondria. Consistenly, MST4 co-localized with the endogenous MAVS in 293 T cells (Fig. 4C). Upon SeV infection, partial distribution of MST4 was detected as dot-like aggregates, which was similar to that of MAVS, indicating that more MST4 was recruited to MAVS upon SeV infection.

MST4 consists of an N-terminal kinase domain (amino acids 1–297) and a C-terminal regulatory domain (amino acids 297–416) (Fig. 4D) [25]. To study the domains responsible for the MST4–MAVS interaction, we generated deletions and truncations of these two proteins and analyzed them by coimmunoprecipitation experiments. MAVS immunoprecipitated with amino acids 1–297 but did not precipitate with amino acids 297–416 (Fig. 4E), indicating that the kinase domain of MST4 was the one involved in the interaction with MAVS. Furthermore, the amino acids 360–540 of MAVS were required for the interaction with MST4 (Fig. 4G). Since the kinase domain of MST4 was involved in its interaction with MAVS, we tested whether this domain was also required for MST4-mediated negative regulation of type I IFN production. We found that it was amino acids 1–297 of MST4 and not the amino acids 297–416 that inhibited the activation of the IFN-β reporter induced by SeV (Fig. 4H). Overall, our results demonstrated that the kinase domain of MST4 was involved in the interaction of MST4–MAVS that negatively regulated the SeV-mediated type I IFN production.

### MST4 impaired the TRAF3/MAVS association

**Fig. 5:**
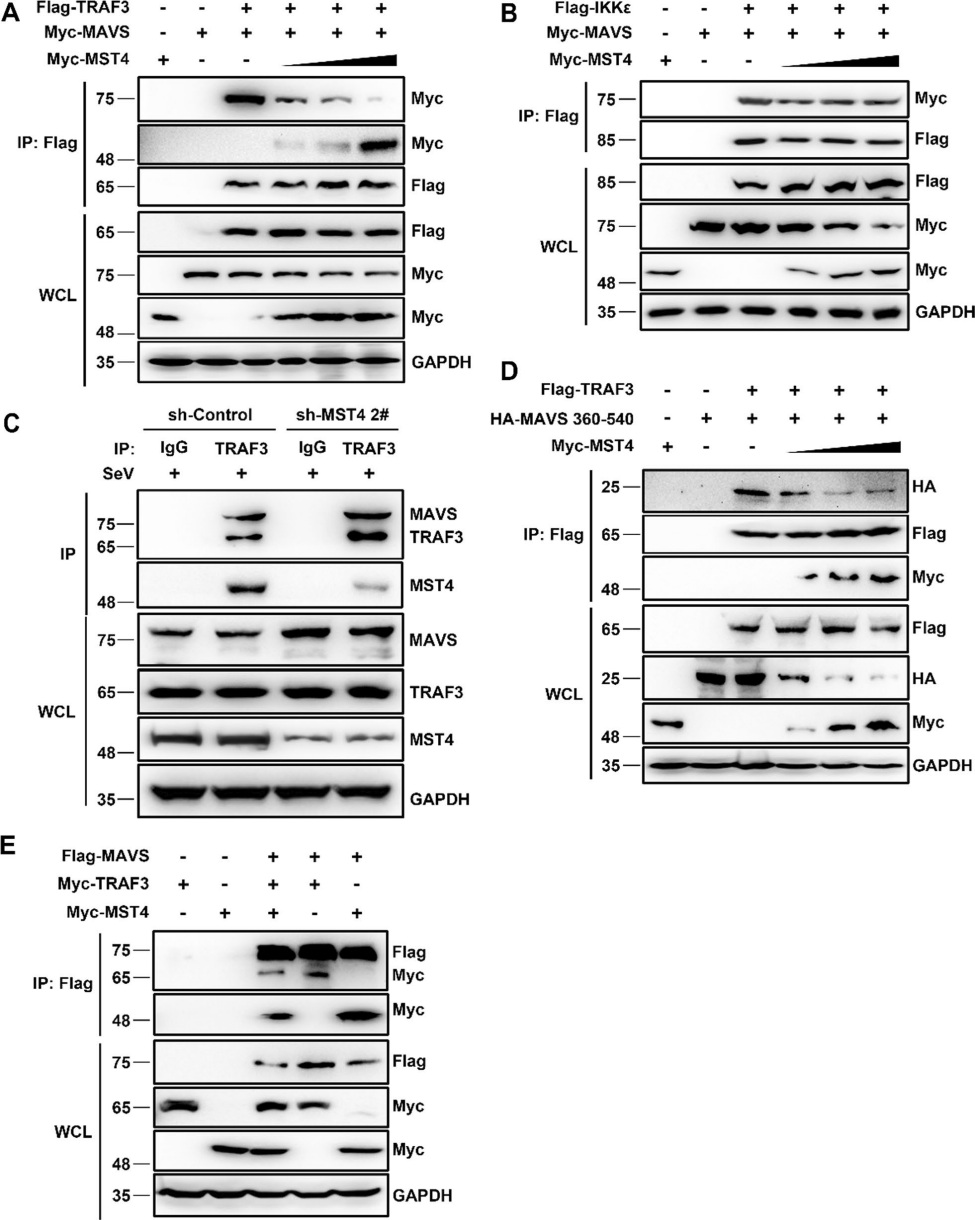
MST4 inhibits the binding of TRAF3 to MAVS. **A** Immunoblot analysis of lysates of 293 T cells transfected with Flag-TRAF3, Myc-MAVS, and increasing amounts of Myc-MST4 for 24 h, followed by immunoprecipitation with anti-Flag beads. **B** Immunoblot analysis of lysates of 293 T cells transfected with Flag-IKKε, Myc-MAVS, and increasing amounts of Myc-MST4 for 24 h, followed by immunoprecipitation with anti-Flag beads. **C** Immunoblot analysis of lysates of 293 T-sh-Control and 293 T-sh-MST4 cells infected with SeV for 8 h, followed by immunoprecipitation with control rabbit-IgG or anti-TRAF3 antibodies. Lysates and immunoprecipitation extracts were probed with MAVS and TRAF3 antibodies. **D** Immunoblot analysis of lysates of 293 T cells transfected with Flag-TRAF3, HA-MAVS 360–540, and increasing amounts of Myc-MST4 for 24 h, followed by immunoprecipitation with anti-Flag beads. **E** Immunoblot analysis of lysates of 293 T cells transfected with Flag-MAVS, Myc-TRAF3, or Myc-MST4 for 24 h, followed by immunoprecipitation with anti-Flag beads. Data information: The data are representative of at least three independent experiments

It is well established that the activity of MAVS depends on its association with members of the TRAF family, such as the E3 ubiquitin ligases TRAF2, TRAF6, and TRAF3 [26]. Moreover, TRAF3 is involved in the activation of the IRF3 pathway, which is essential for the induction of IFNs [27]. Thus, we determined the effect of MST4 on the MAVS/TRAF3 association. We also performed the coimmunoprecipitation studies of MAVS and TRAF3 in the presence of increasing amounts of MST4 and compared it to the known association between MAVS and IKKε, which was used as a control [28]. As shown in Fig. 5A, B, increasing amounts of MST4 led to a strong reduction of MAVS/TRAF3 association but showed no effect on the MAVS/IKKε association. Furthermore, we observed that MST4 could inhibit the endogenous interaction between MAVS and TRAF3 upon SeV infection (Fig. 5C). TRAF3 has also been associated with the 450–468 domain of MAVS [27]. Further, we analyzed the effect of increasing concentrations of MST4 on the association of TRAF3 with the 360–540 construct of MAVS, which also contained one of the MST4 binding domains. Interestingly, MST4 strongly abolished the interaction between TRAF3 and the 360–540 domain of MAVS (Fig. 5D).

Moreover, we determined the possible mechanism by which MST4 inhibited the MAVS/TRAF3 association and speculated that MST4 might compete with TRAF3 to interact with MAVS. The coimmunoprecipitation results confirmed our hypothesis, where the reduction in the TRAF3–MAVS interaction was observed in the presence of MST4 while in the presence of TRAF3, the reduction was observed in the MST4–MAVS interaction (Fig. 5E). Overall, our findings revealed that MST4 competed with TRAF3 to bind to the 360–540 domain of MAVS, thereby inhibiting the TRAF3/MAVS association.

### MST4 destabilizes MAVS by facilitating the Smurf1-mediated K48-linked ubiquitination of MAVS

**Fig. 6:**
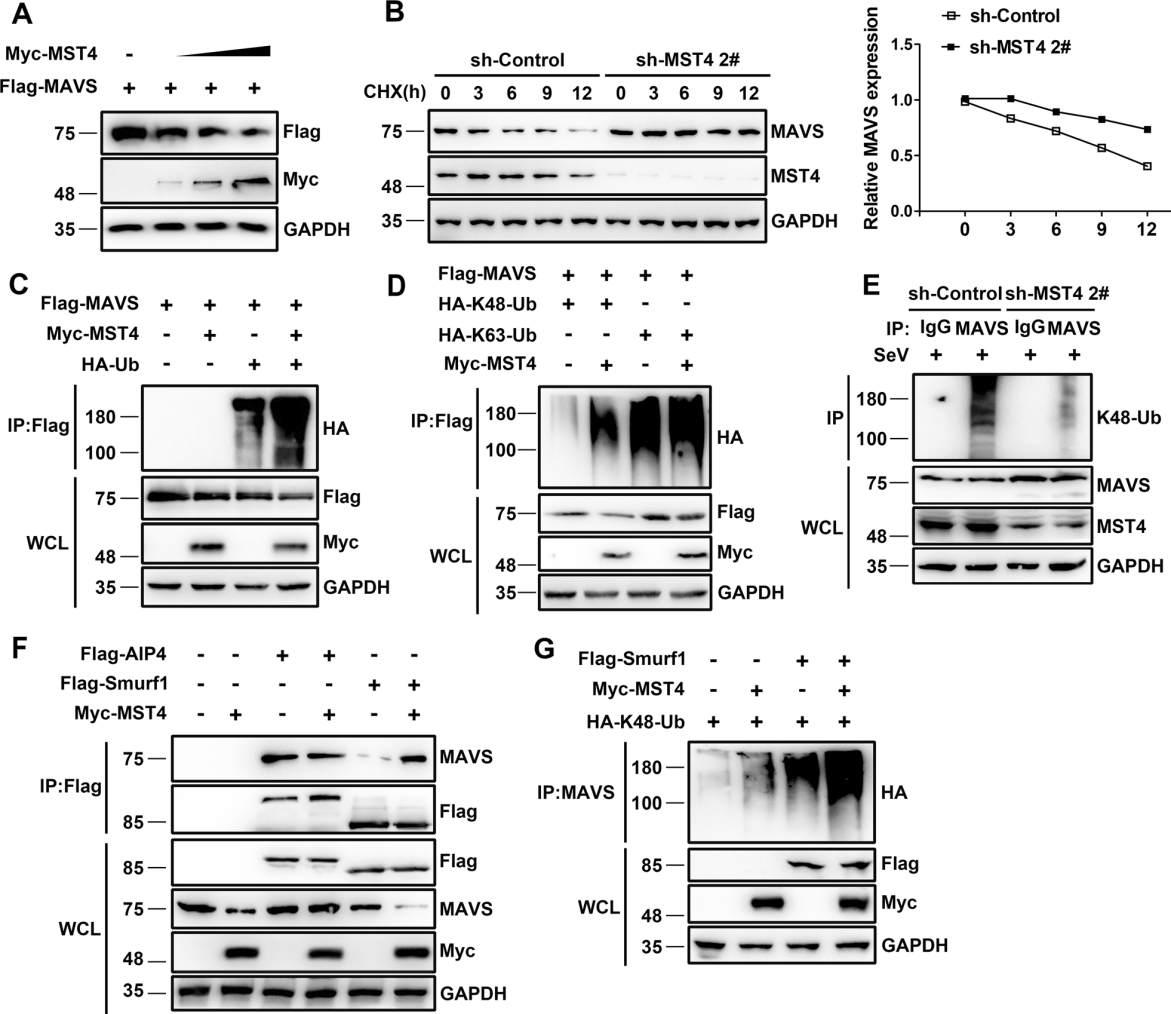
MST4 facilitates K48-linked ubiquitination of MAVS. **A** Immunoblot analysis of lysates of 293 T cells transfected with Flag-MAVS and increasing amounts of Myc-MST4 for 24 h. **B** Immunoblot analysis of lysates of 293 T-sh-Control and 293 T-sh-MST4 cells incubated with 100 µg/mL CHX at indicated durations (left). The quantification of the relative expression levels of MAVS (right). **C** Immunoblot analysis of lysates of 293 T cells transfected with Flag-MAVS, Myc-MST4, or HA-Ub for 24 h, followed by immunoprecipitation with anti-Flag beads. **D** Immunoblot analysis of lysates of 293 T cells transfected with various combinations of plasmids for 24 h, followed by immunoprecipitation with anti-Flag beads. **E** Immunoblot analysis of lysates of 293 T-sh-Control and 293 T-sh-MST4 cells infected with SeV for 8 h, followed by immunoprecipitation with control rabbit-IgG or anti-MAVS antibodies. Lysates and immunoprecipitation extracts were probed with K48-Ub, MAVS, and MST4 antibodies. **F** Immunoblot analysis of lysates of 293 T cells transfected with Flag-AIP4, Flag-Smurf1, or Myc-MST4 for 24 h, followed by immunoprecipitation with anti-Flag beads. **G** Immunoblot analysis of lysates of 293 T cells transfected with Flag-Smurf1 or Myc-MST4 and HA-K48-Ub for 24 h, followed by immunoprecipitation with anti-MAVS antibody. Data information: The data are representative of at least three independent experiments

Interestingly, MST4 expression in 293 T cells promoted protein degradation of MAVS (Fig. 6A) but barely changed its mRNA levels (Additional file 1: Fig. S4A). Next, the 293 T cells were treated with cycloheximide (CHX) for 6 h to inhibit the protein synthesis. The endogenous MAVS was detected at various time points in the presence or absence of MST4, and it was found that an ample number of MAVS was retained after the addition of cycloheximide in the absence of MST4 (Fig. 6B). These data indicated that MST4 played a key role in destabilizing MAVS.

Multiple lines of evidence have revealed that the degradation of MAVS is controlled by ubiquitin-meditated proteolysis [21, 29]. Hence, we further investigated the effects of MST4 on MAVS ubiquitination. The coimmunoprecipitation results showed that the ubiquitination of MAVS was enhanced in the presence of MST4 (Fig. 6C). Additionally, MST4 was observed to facilitate the K48-linked and not K63-linked ubiquitination of both exogenous and endogenous MAVS (Fig. 6D, E).

Since E3 ubiquitin ligases, Smurf1 and AIP4, both have been shown to mediate K48-ubiquitination and degradation of MAVS [29, 30], it is important to identify the specific ubiquitin ligase involved in the MST4-regulated MAVS ubiquitination. Therefore, we investigated whether it was Smurf1- or the AIP4-mediated MAVS, whose stability and ubiquitination were regulated by the MST4. It was found that the interaction between Smurf1 and MAVS was increased in the presence of MST4 while the level of MAVS protein was considerably decreased in Smurf1 and MST4 co-expressing cells compared to the cells transfected only with Smurf1. This suggested that MST4 inhibited Smurf1-mediated degradation of MAVS (Fig. 6F). According to the stability assay, the ubiquitination of MAVS was enhanced in Smurf1 and MST4 co-expressing cells compared to those transfected with only Smurf1 (Fig. 6G). Collectively, our results indicated that MST4 facilitated the interaction between the E3 ubiquitin ligase Smurf1 and MAVS while promoting K48-linked ubiquitination of MAVS, thereby accelerating the ubiquitin-mediated proteasome degradation of MAVS.

### MST4 kinase activity is essential in the inhibition of RLR signaling

**Fig. 7:**
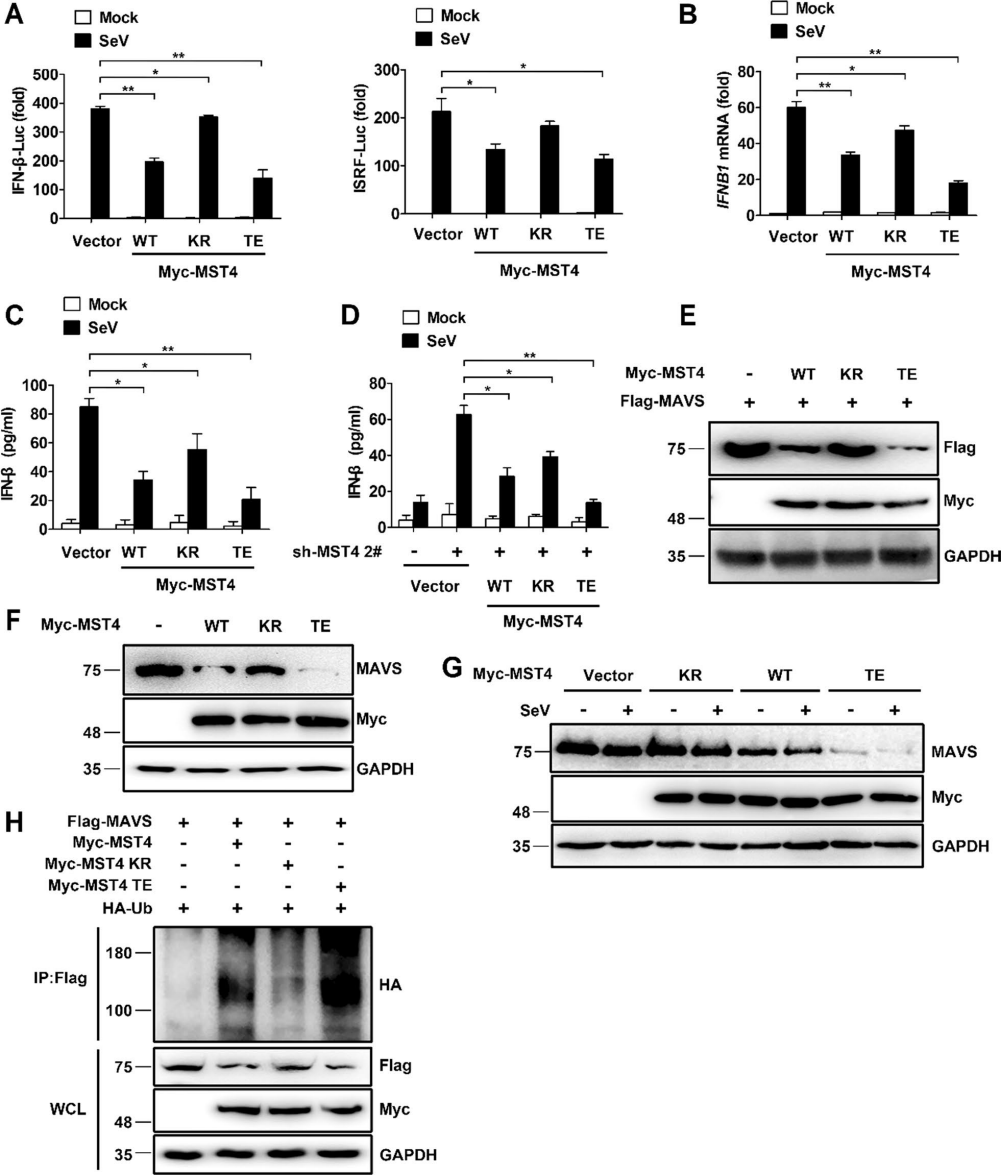
The suppression of RLR signaling by MST4 is dependent on its kinase activity. **A** Luciferase activity of lysates of 293 T cells transfected with empty vector or vector expressing Myc-tagged wild-type MST4 (WT) or MST4 with no kinase activity (KR) or constitutively activated kinase activity (TE) along with IFN-β-Luc or ISRE-Luc for 24 h, and then treated with SeV for 8 h. **B** Quantitative PCR analysis of *IFNB1* mRNA in 293 T cells transfected with empty vector or Myc-MST4-WT, Myc-MST4-KR, or Myc-MST4-TE for 24 h, and then treated with SeV for 8 h. **C** Secretion of IFN-β by 293 T cells transfected with empty vector or Myc-MST4-WT and Myc-MST4-KR or Myc-MST4-TE for 24 h, and then treated with SeV for 12 h. **D** Secretion of IFN-β by 293 T-sh-MST4 cells transfected with various combinations of plasmids for 24 h, and then treated with SeV for 12 h. **E** Immunoblot analysis of lysates of 293 T cells transfected with Myc-MST4-WT, Myc-MST4-KR or Myc-MST4-TE, and Flag-MAVS for 24 h. **F** Immunoblot analysis of lysates of 293 T cells transfected with Myc-MST4-WT, Myc-MST4-KR, or Myc-MST4-TE for 24 h. **G** Immunoblot analysis of lysates of 293 T cells transfected with various combinations of plasmids for 24 h and then infected with SeV for 8 h. **H** Immunoblot analysis of lysates of 293 T cells transfected with Myc-MST4-WT, Myc-MST4-KR or Myc-MST4-TE, Flag-MAVS, and HA-Ub for 24 h, followed by immunoprecipitation with anti-Flag beads. Data information: The data are represented as mean ± SD (**A**–**D**: n = 3). *P < 0.05 and **P < 0.01 (unpaired two-tailed Student’s *t*-test). The data are representative of at least three independent experiments

Since MST4 is a kinase, we next sought to determine whether its inhibitory effect on the SeV-induced immune responses was dependent on its kinase activity or not. Hence, we used two MST4 mutants whose kinase activity was either constitutively activated (substitution of glutamic acid instead of threonine at position 178) or completely abolished (substitution of arginine instead of lysine at position 53). The expression of the constitutively activated mutant of MST4 (TE) in 293 T cells resulted in greater inhibition of SeV-stimulated IFN-β and ISRE-responsive luciferase activity (Fig. 7A) along with the transcription and secretion of *IFNB1* and IFN-β, respectively, (Fig. 7B, C) compared to the expression seen in the wild-type (WT) MST4. In contrast, the expression of the MST4 mutant lacking kinase activity abrogated the inhibition of SeV-induced production of IFN-β by MST4 (Fig. 7A–C). Depletion of endogenous MST4 promoted the secretion of IFN-β in 293 T cells (Fig. 7D). After the treatment with the shRNA, ‘rescue’ of the cells by transfection was performed to express the constitutively active mutant of MST4, which inhibited the SeV-induced IFN-β production to a greater extent than observed in the transfection of cells done to express MST4-WT. However, the transfection of cells performed to express the MST4-mutant lacking kinase activity did not alter the production of IFN-β (Fig. 7D).

Given that MST4 plays a key role in destabilizing MAVS, we investigated whether MST4 could also promote the degradation of MAVS via its kinase activity. The Western blot results showed that constitutively activated mutant of MST4 accelerated greater degradation of both exogenous and endogenous MAVS compared to the MST4-WT with or without SeV infection (Fig. 7E–G). Consistent with these results, we observed that ubiquitination of MAVS was increased upon transfection of MST4-TE into 293 T cells compared to that of the MST4-WT (Fig. 7H). Overall, these results suggested that the kinase activity of MST4 was required for the negative regulation of the RLR-mediated IFN signaling pathway.

## Discussion

RLRs are the key sensors of virus infection that mediate the induction of genes encoding interferons, inflammatory cytokines, and interferon-related molecules [31, 32]. However, the production of type I interferon and proinflammatory cytokines must be tightly regulated since the aberrant production of type I interferon can be deleterious to the host [33]. A previous study demonstrated that MST4 kinase could directly phosphorylate TRAF6 to confine macrophage-mediated inflammation, thereby avoiding tissue damage caused by immune overreaction [18]. Recently, MST4 was reported to modulate the neuro-inflammatory response by regulating the IκBα signaling pathway, which affected the early outcome of experimental ischemic stroke in mice [34]. Additionally, Wu et al. reported that MST4 attenuated the NLRP3 inflammasome-mediated neuroinflammation, affecting the prognosis after intracerebral hemorrhage in mice [35]. In this study, we found that MST4 functioned as a negative regulator of the RLR-mediated type I IFN production.

Using the luciferase reporter assay system, we found that MST4 inhibited the IFN-β production induced by Poly (I:C) transfection and SeV infection, whereas the knockdown of MST4 expression potentially activated the IFN-β promoter triggered by Poly (I:C) transfection and SeV infection. Moreover, the mRNA levels of *IFNB1*, *IFIT1*, and *IFIT2,* along with the levels of secreted IFN-α and IFN-β were significantly increased upon knockdown of MST4 stimulated by Poly (I:C) transfection or SeV. These results showed that MST4 could act as a negative regulator of RLR-mediated type I IFN production.

MAVS has been identified as an essential adaptor protein for controlling RLR independent cytosolic signaling and the production of type I IFNs [36, 37]. A reporter assay further indicated that MST4 greatly impaired the activity of the IFN-β promoter, which was induced by RIG-I-N, MDA5-N, and MAVS. Coimmunoprecipitation experiments indicated that it was MAVS and not RIG-I and MDA5 that were associated with MST4. Also, double immunofluorescence staining revealed that endogenous MST4 showed a similar localization pattern as MAVS. Multimerization or aggregation has been demonstrated as a key feature of MAVS antiviral function [38]. Prion-like aggregation of MAVS is required for its activation, which presumably releases its autoinhibition on the amino acids 360–540 of MAVS after a viral infection [39, 40]. In the present study, we found that the amino acids 360–540 of MAVS were required for interaction with MST4, suggesting a role of MST4 in the production of type I IFN by modulating prion-like aggregation of MAVS. Meanwhile, MAVS immunoprecipitated with N-terminal kinase domain (amino acids 1–297). Furthermore, MST4 kinase activity is essential destabilizing MAVS. We speculated that MST4 may phosphorylate MAVS, which promotes its ubiquitin-mediated degradation by E3 ligases Smurf1. A detailed further study is an interesting future direction.

Upon sensing the cytosolic RNA, RLRs activate and induce MAVS to form prion-like aggregates [26]. The recruitment of TRAFs, including TRAF2, TRAF3, TRAF5, and TRAF6, is crucial to the complex formation of MAVS and the downstream signaling [26, 41]. Interaction of MAVS with TRAF2 or TRAF6 is involved in IKK-dependent NF-κB activation, whereas its interaction with TRAF3 is specifically involved in the TBK1-dependent IRF3 activation [42]. Moreover, TRAF3 binds to amino acids 450–468 of MAVS to regulate downstream signaling of type I IFN [42, 43]. Here, we found that MST4 interacted specifically with the C-terminal of the TRAF3-binding site of MAVS (aa 455–460) and then overlapped the TRAF3 binding site within the MAVS. Consequently, we observed that MST4 competes with TRAF3 to bind to the 360–540 domain of MAVS, thereby inhibiting the TRAF3/MAVS association, which resulted in the inhibition of IFN production and cellular antiviral responses.

To maintain the antiviral innate immune homeostasis, MAVS is degraded by ubiquitin-mediated proteolysis to turn off the production of type I IFN during the later phases of viral infection [44]. The Trim25 and AIP4 ubiquitin E3 ligases have been reported to mediate the viral-triggered ubiquitination and degradation of MAVS [29, 45]. In this study, we found that MST4 promoted K48-linked polyubiquitination and degradation of MAVS, providing another layer of molecular intricacy of how MST4 modulates the RLR pathway. Importantly, we also showed that this process is dependent on the E3 ligase, Smurf1. Also, PCBP2 is reported to function as an adaptor that recruits MAVS to AIP4, leading to greater degradation of MAVS [29]. Thus, our data added MST4 to the growing list of AIP4 adaptors as another member. More intriguingly, MST4 might also have a role in modulating IRF3 or IKK after SeV infection. Our data indicated that MST4 suppressed the nuclear translocation of IRF3 and affected the stability of IRF3 and IKK. We speculate that MST4 may regulate the phosphorylation or ubiquitination process of IRF3 or IKK, leading to regulating IRF3 nuclear translocation and stability of IRF3 or IKK. This is a tempting hypothesis, which is an interesting future direction.

## Conclusion

In conclusion, we provided evidence that MST4 acted as a critical negative regulator of the production of type I IFN by disrupting MAVS–TRAF3 complex formation along with promoting E3 ligase Smurf1-mediated MAVS ubiquitination for degradation. Understanding these processes may shed light on MST4 function in the RLR-mediated type I IFN production, providing new complexity to the layers of MAVS regulation.

## Supplementary Information

**Additional file 1.** Supplementary Figures (Figure S1–S4) and Table 1.
