# Supplementary material for: MST4 negatively regulates type I interferons production via targeting MAVS-mediated pathway
Source: Cell Commun Signal. 2022 Jul 12;20:103. doi: 10.1186/s12964-022-00922-3 (PMC9274187; doi:10.1186/s12964-022-00922-3)
Supplement: Supplementary file 2 — Additional file 1. Supplementary Figures (Figure S1–S4) and Table 1. [file 12964_2022_922_MOESM2_ESM.docx]

Additional file 1 for

MST4 Negatively Regulates type I interferons production via targeting MAVS-mediated pathway

Wei Liu^1*^, Zhenling Ma^1^, Yaru Wu^1^, Cui Yuan^1^, Yanyan Zhang^1^, Zeyang Liang^1^, Yu Yang^1^, Wenwen Zhang^1^, Pengtao Jiao^2*^

^1^College of Life Sciences, Henan Agricultural University, 450002 Zhengzhou, China； ^2^CAS Key Laboratory of Pathogenic Microbiology and Immunology, Institute of Microbiology, Chinese Academy of Sciences, 100101 Beijing, China

^*^Correspondence: [liuv@henau.edu.cn](mailto:liuv@henau.edu.cn); [pengtaojiao66@126.com](mailto:pengtaojiao66@126.com)

**This PDF file includes:**

Figures. 1 to 4

Table 1


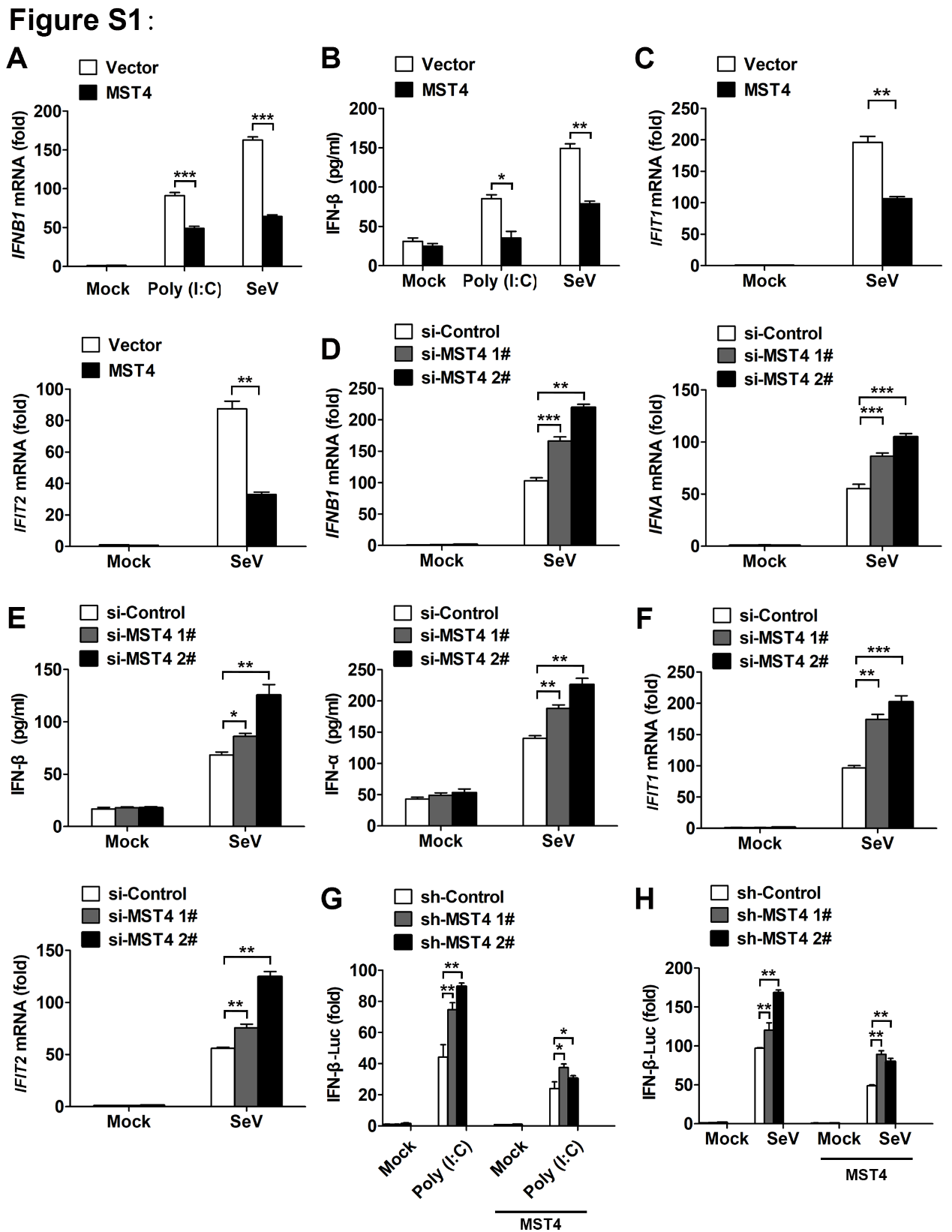


**Additional file 1: Fig. 1** MST4 negatively regulates RLR-mediated type I IFN production. (**A**) Quantitative PCR analysis of *IFNB1* mRNA levels in 293T cells transfected with Myc-MST4, and then transfected with Poly (I:C) or infected with SeV for 8 h. (**B**) ELISA of IFN-β production in the supernatants of 293T cells transfected with Myc-MST4, and then transfected with Poly (I:C) or infected with SeV for 12 h. (**C**) Quantitative PCR analysis of *IFIT1* and *IFIT2* mRNA levels in 293T cells transfected with Myc-MST4, and then treated with SeV for 8 h. (**D**) Quantitative PCR analysis of *IFNB1* and *IFNA* mRNA levels in THP-1 cells transfected with MST4 siRNA or scrambled siRNA for 48 h and then treated with SeV for 8 h. (**E**) ELISA of IFN-β and IFN-α production in the supernatants of THP-1 cells transfected with MST4 siRNA or scrambled siRNA for 48 h, and then treated with SeV for 12 h. (**F**) Quantitative PCR analysis of *IFIT1* and *IFIT2* mRNA levels in THP-1 cells transfected with MST4 siRNA or scrambled siRNA for 48 h and then treated with SeV for 12 h. (**G, H**) Luciferase activity of lysates in 293T-sh-Control and 293T-sh-MST4 cells transfected for 24 h with IFN-β-Luc, along with empty vector or Myc-MST4, and then transfected with Poly (I:C)(**G**) or infected with SeV (**H**) for 8 h. The data are represented as mean ±SD (**A-H**: n = 3). **P* < 0.05, ***P* < 0.01, and ****P* < 0.001 (unpaired two-tailed Student’s *t*-test). The data are representative of at least three independent experiments.


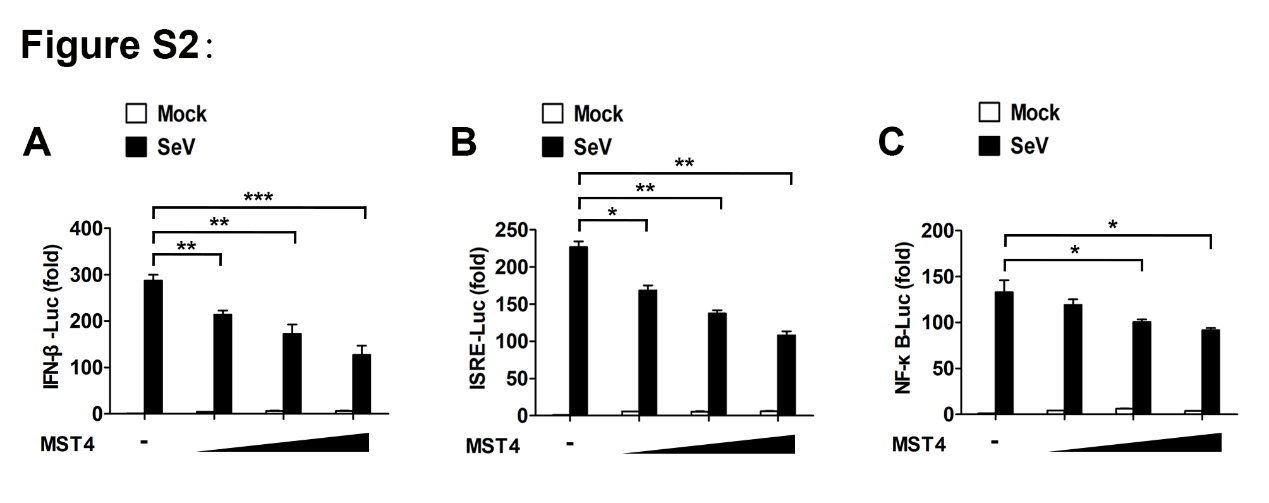


**Additional file 1: Fig. 2** MST4 deficiency enhances IRF3 activation. (**A-C**) Luciferase activity of lysates in 293T cells transfected for 24 h with IFN-β-Luc (**A**), NF-κB-Luc (**B**), or ISRE-Luc (**C**), and then treated with SeV for 8 h.


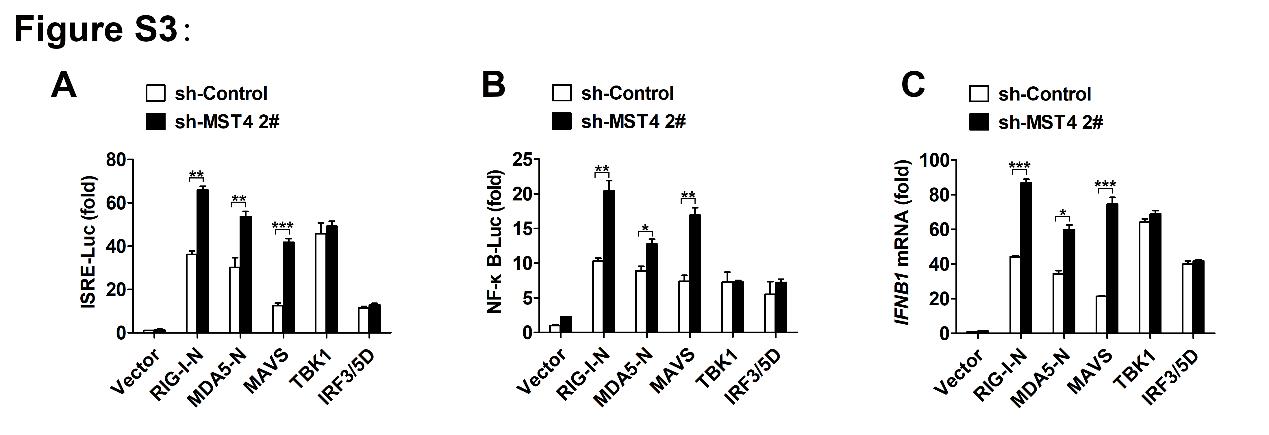


**Additional file 1: Fig. 3** MST4 inhibits type I IFN production by targeting MAVS. (**A, B**) Luciferase activity of lysates in 293T-sh-Control and 293T-sh-MST4 cells transfected for 24 h with ISRE-Luc (**A**), or NF-κB-Luc (**B**), along with Flag-tagged RIG-I-N, MDA5-N, MAVS, TBK1, or IRF3/5D, and then treated with SeV for 8 h. (**C**) Quantitative PCR analysis of *IFNB1* mRNA levels in 293T-sh-Control and 293T-sh-MST4 cells transfected with Flag-tagged RIG-I-N, MDA5-N, MAVS, TBK1, or IRF3/5D for 24 h and then treated with SeV for 8 h.


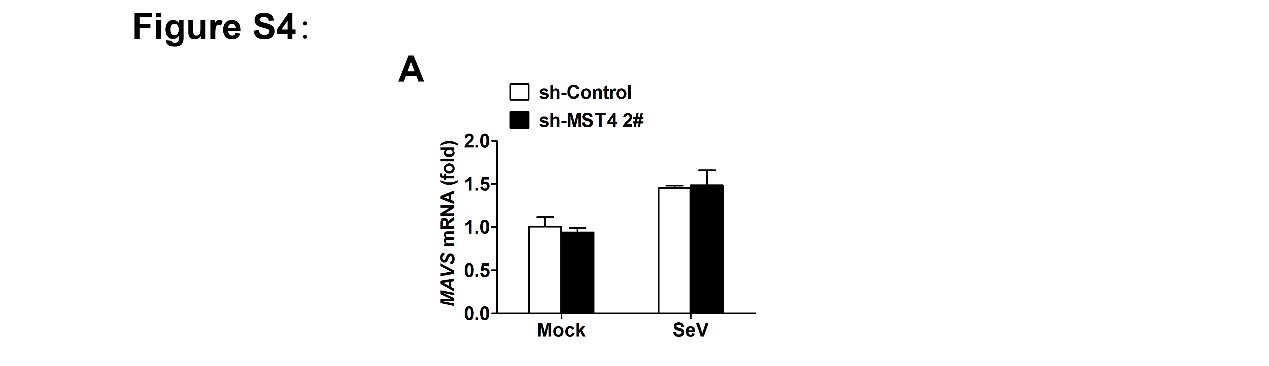


**Additional file 1: Fig. 4** MST4 inhibits type I IFN production by targeting MAVS. (**A**) Quantitative PCR analysis of *MAVS* mRNA levels in 293T-sh-Control and 293T-sh-MST4 cells treated with SeV for 8 h.

**Additional file 1: Table 1.** List of primers used in the study.

| **Gene name** | **Primers** | **Sequence (5’-3’)** |
| --- | --- | --- |
| *IFNB1* | Forward | 5’-AACTGCAACCTTTCGAAGCC-3’ |
|  | Reverse | 5’-TGTCGCCTACTACCTGTTGTGC-3’ |
| *IFIT1* | Forward | 5’-TCATTTTGCATCCCATAGGAGGTT-3’ |
|  | Reverse | 5’-GACTTTGGTCCCCCAGCTTT-3’ |
| *IFIT2* | Forward | 5’-GCCGCATCGCCGTCTCCTAC-3’ |
|  | Reverse | 5’-CTCCAGGGCTTCATTCATAT-3’ |
| *MAVS* | Forward | 5’-GAAATTATTCCTGCAAGCCAATTT-3’ |
|  | Reverse | 5’-TCACCCTTCTTTTTCATGTAG-3’ |
| *GAPDH* | Forward | 5’-TTGTCTCCTGCGACTTCAACAG-3’ |
|  | Reverse | 5’-GGTCTGGGATGGAAATTGTGAG-3’ |
